# Brazilian Public Health System protocol for the diagnosis, treatment, and clinical monitoring of acute ischemic stroke

**DOI:** 10.1055/s-0045-1806923

**Published:** 2025-06-20

**Authors:** Aline Rocha, Ana Carolina Pereira Nunes Pinto, Cinara Stein, Verônica Colpani, Débora Gräf, Gilson Dorneles, Karlyse Claudino Belli, Suena Parahiba, Maicon Falavigna, Sheila Ouriques Martins, Álvaro Nagib Atallah

**Affiliations:** 1 Cochrane Brasil, Núcleo de Avaliação de Tecnologias em Saúde, São Paulo SP, Brazil.; 2 Universidade Federal de São Paulo, Disciplina de Medicina de Urgência e Medicina Baseada em Evidências, São Paulo SP, Brazil.; 3 Institut de Recerca de l'Hospital de la Santa Creu i Sant Pau (IR Sant Pau), Barcelona, Catalunha, Spain.; 4 Centro Cochrane Iberoamericano, Barcelona, Catalunha, Spain.; 5 Hospital Moinhos de Vento, Escritório de Projetos, Responsabilidade Social, Programa de Apoio ao Desenvolvimento Institucional do Sistema Único de Saúde, Porto Alegre RS, Brazil.; 6 Universidade Federal do Rio Grande do Sul, Hospital de Clínicas de Porto Alegre, Porto Alegre RS, Brazil.; 7 HTA Unit, Inova Medical, Porto Alegre RS, Brazil.

**Keywords:** Ischemic Stroke, Clinical Protocols, Tissue Plasminogen Activator, Thrombectomy, Public Health

## Abstract

The Brazilian Ministry of Health has issued new recommendations for the management of patients with acute ischemic stroke. The protocol covers aspects related to diagnosis, treatment, and patient monitoring, expanding the available treatment options for patients treated within the Brazilian Public Health System (Sistema Único de Saúde – SUS, in Portuguese). This article discusses some of the recommendations presented in the current Clinical Practice Protocol, aiming to spread awareness of the document's updates and assist healthcare professionals and services in implementing these recommendations.

## INTRODUCTION

Stroke ranks among the leading causes of disability and mortality globally. 1 2 Annually, it affects 15 million individuals worldwide, leading to 5.5 million deaths and leaving an additional 5 million people with permanent disabilities. This constitutes a substantial public health burden. 3 This disease is primarily observed in middle-aged and older adults. 3 Findings from a Brazilian national prospective study revealed an annual incidence of 108 cases per 100,000 inhabitants. 4

Strokes are categorized into ischemic (resulting from arterial obstruction with subsequent alteration of cerebral blood flow), intracerebral hemorrhage (a nontraumatic focal accumulation of blood within the cerebral parenchyma or ventricular system), and subarachnoid hemorrhage. Ischemic strokes are the most common, accounting for 75 to 85% of all stroke cases. 1 4 5 Consequently, effective management of modifiable risk factors in primary care settings can play a crucial role in preventing most strokes. Moreover, timely and efficient treatment is pivotal in averting long-term disabilities and fatalities.

Given its prevalence, clinical condition, and impact on patients' quality of life and functional capacity, stroke poses a significant financial challenge to healthcare systems, affecting not only the patients but also their families and caregivers. 6 Over the last two decades, its global burden has intensified. 4 In 2015, ischemic heart disease and stroke were the top two causes of premature death worldwide and the primary contributors to disability-adjusted life years (DALYs) across 119 countries and territories. 7 Thus, there is considerable public health interest in identifying safe, effective, and affordable therapies for prevention and treatment. 8

In Brazil, Clinical Protocols and Therapeutic Guidelines are the official documents of the Brazilian Public Health System (SUS) that establish criteria for the diagnosis of a health condition, recommended treatment, clinical control mechanisms, and follow-up of therapeutic outcomes for SUS stakeholders. 9

The Brazilian Ministry of Health (MoH) has recently released updated recommendations on clinical protocols for the management of acute ischemic stroke (AIS), 10 which update the previous Clinical Protocol for Thrombolysis in AIS. This revision was motivated by the availability of new stroke treatment technologies within the SUS, ensuring access for the entire Brazilian population covered by this system. Moreover, the updated protocol aims to establish a more assertive and multidisciplinary healthcare model, focusing on secondary and tertiary care, for diagnosing and treating AIS, through both pharmacological and nonpharmacological interventions. In the following sections, we will outline the methodology employed to update the protocol and the panelists' recommendations. 10

## METHODS

The development process of the MoH's protocol 10 adhered to the Methodological Guideline for the Development of Clinical Protocols, 9 advocating the use of the Grading of Recommendations Assessment, Development and Evaluation (GRADE) system. 11 This framework categorizes the certainty of evidence from the existing literature into four levels: very low, low, moderate, and high. According to this methodology, recommendations can be classified as strong or weak (conditional), in favor or against the intervention. 11

The target audience includes healthcare professionals engaged in treating patients with AIS, particularly neurologists, neuroradiologists, neuro-intensivists, and specialists in emergency medicine, emergency surgery, and vascular surgery. The protocol is aimed at patients aged 18 and older diagnosed with AIS.

To ensure that the protocol is updated periodically, it must be reviewed, ideally with the involvement of the original authors. For protocols within SUS, current legislation requires updates every 2 years, or whenever new technologies are incorporated and significant changes in practice related to the protocols become necessary. 9

### Organization, panel composition, and protocol development

The protocol development group comprised a panel of experts coordinated by the Department of Management and Incorporation of Health Technologies and Innovation of the Bureau of Science, Technology, Innovation and Strategic Health Supplies (Departamento de Gestão e Incorporação de Tecnologias e Inovação em Saúde of Secretaria de Ciência, Tecnologia, Inovação e Insumos Estratégicos em Saúde of the Brazilian MoH - DGITIS/SCTIE/MS, in Portuguese). The panel of experts included emergency medicine physicians, vascular surgeons, neurologists, neuro intensivists, neuroradiologists, and representatives from the MoH, centers of excellence, medical societies (Brazilian Stroke Society, Brazilian Academy of Neurology, and Brazilian Society of Interventional Neuroradiology), universities and hospital members, and representatives from nonprofit associations (Rede Brasil AVC).

The organization of the protocol was initiated on March 30, 2021, with a meeting to define its scope. This meeting determined the clinical questions that would be addressed in the MoH document. The drafting group and the manager committee held two online videoconferences with the experts and relevant stakeholders to present the evidence syntheses and develop the recommendations that would be included (on October 29 and November 5, 2021), based on the GRADE evidence to decision (EtD) framework for health system and public health decisions, as well as considering the level of priority of the problem, desirable effects, undesirable effects, certainty of evidence, balance of effects, required resources, acceptability, and feasibility.

The protocol development process actively involved stakeholders, including societies, universities, member hospitals, and nonprofit associations. This involvement took place through meetings with experts, medical association and patient representatives, and methodologists in scoping meetings, to present the new evidence for the update and the clinical questions that would guide this update. At this point, patient representatives were also involved in prioritizing outcomes, thus ensuring that the questions and treatments evaluated are aligned with what patients value most. The panel of experts was responsible for evaluating the literature evidence regarding the prioritized questions in the scope document and for developing their respective recommendations.

For this protocol, diagnostic and monitoring questions were not prioritized, although they are part of the Guideline Development Group (GL) scope. This means that a systematic review was not performed for these aspects, and that the questions already in place on these themes remained the same for the protocols. The members of the drafting group and the managing committee did not interfere in the decision-making process for the panelists' development of the recommendations. Upon completion of the recommendations and the finalization of the update, the protocol was submitted for public consultation, allowing the community, including patients, to provide input.

These contributions were reviewed by the drafting committee and subsequently forwarded to the Brazilian Commission for the Incorporation of Health Technologies into the Unified Health System (Comissão Nacional de Incorporação de Tecnologias no Sistema Único de Saúde - CONITEC, in Portuguese), which evaluated the recommendations and validated the published version of the clinical protocol.

All voting members and methodologists of the drafting group disclosed any potential conflicts of interest using the Ministry of Health's standard declaration. Individuals with significant conflicts related to specific issues in the document were excluded from participating in discussions on those topics. The most commonly reported conflict of interest among panel members was the receipt of fees for services rendered to institutions with a vested interest in the protocol's scope. The entire process followed the MoH's guidelines. 9

### Identification of clinical question

The methodology defined in the MoH Development Manual, as recommended by the Brazilian MoH, was used for the identification and development of research questions. This methodology indicates that the drafting group should conduct a preliminary literature review and propose questions to the panelists that could be included in the scope of the protocol. To recommend the questions, the following criteria were used: the availability of the medication for prescription in Brazil, the indication in the specific medication label for use in AIS, and a relevant clinical doubt regarding its use.

The process of updating the protocol recommended the evaluation of the following clinical questions:

- Should alteplase be administered to patients with or without large vessel occlusion and AIS symptoms within 4.5 hours?
- Should alteplase be administered to patients with symptom onset > 4.5 hours?
- Should alteplase be administered to patients with unknown symptom onset or wake-up stroke?
- Should tenecteplase be used as an alternative to thrombolysis?
- Should mechanical thrombectomy be performed in patients with large vessel occlusion (LVO) and symptom onset < 8 hours?
- Should mechanical thrombectomy be performed in patients with LVO and a symptom onset window greater than 8 hours but less than 24 hours?
- Should mechanical thrombectomy be performed in patients with LVO and an indeterminate symptom onset time or a wake-up stroke?
- Should general anesthesia or conscious sedation be used for patients undergoing mechanical thrombectomy?

### Search and evidence synthesis

For each research question, a structured search was conducted in the following databases: Medline/PubMed, EMBASE, Cochrane Central, LILACS, and Epistemonikos. Searches were also performed in clinical guideline repositories to identify potential updates regarding the management of patients with ischemic stroke. The search strategies were executed in July 2021.

A detailed description of the methodology (including the complete search strategy, screening and study selection, data collection and analysis, risk of bias, and certainty of evidence assessment), evidence synthesis, and additional justification for the judgment were comprehensively outlined in the document published by the MoH (available in XX).

In summary, article selection was performed according to pre-established eligibility criteria for each research question. Generally, systematic reviews with meta-analysis (SRMA) were selected, and in the absence of high-quality SRMA, randomized controlled trials (RCTs) were considered. Assessments were independently conducted by two researchers, with any discrepancies resolved through consultation with a third evaluator.

The quality of systematic reviews was evaluated using the ROBIS tool. Randomized controlled trials were assessed using the Risk of Bias tool proposed by Cochrane (ROB 2.0). 12 A standardized data extraction form developed by the drafting group was used for data extraction. The results are presented as relative risk (RR) or mean difference (MD), with their respective 95% confidence intervals (CIs). The random-effects model was employed in the meta-analyses.

### Assessing certainty of evidence and developing recommendations

For the development of recommendations, the findings were summarized by the drafting group, considering the following aspects: benefits, risks, CoE, costs, feasibility, and other considerations (equity, acceptability, and patient preference). The GRADE system was used to assess CoE, which is classified into four levels: high, moderate, low, and very low ( Table 1 ). 9 13 All assessments were conducted independently by two researchers, with any disagreements resolved through consultation with a third evaluator.

**Table 1:** Levels of evidence according to the GRADE system

Level	Definition	Implications
**High**	Strong confidence that the true effect is close to the estimate.	It is unlikely that additional works will change the confidence in the effect estimate.
**Moderate**	Moderate confidence in the estimated effect.	Future works may change the confidence in the effect estimate, or even the estimate.
**Low**	Confidence in the effect is limited.	Future works are likely to have a major impact on the confidence in the effect estimate.
**Very low**	Confidence in the effect estimate is very limited. There is an important degree of uncertainty in the findings.	Any effect estimate is uncertain.

Abbreviation: GRADE, Grading of Recommendations Assessment, Development and Evaluation.

Source: Diretrizes metodológicas: Sistema GRADE – Manual de graduação da qualidade da evidência e força de recomendação para tomada de decisão em saúde/Ministério da Saúde, Secretaria de Ciência, Tecnologia e Insumos Estratégicos, Departamento de Ciência e Tecnologia. 13

For each recommendation, during the protocol development process, the course of action (whether to carry out the proposed action or not) and the strength of the recommendation were discussed. The strength was defined as either strong (the group is highly confident that the benefits outweigh the risks) or conditional (there are still uncertainties about the balance between benefit and risk), according to the GRADE system ( Table 2 ). Additional statements about the recommendations, such as possible exceptions to the proposed actions or clarifications, were documented throughout the published clinical protocol. 10 The direction and strength of the recommendation, as well as its wording, were defined during the recommendation meetings. The terms “we recommend” and “we suggest” denote different degrees of emphasis on the strength of the recommendation, as illustrated below:

- “We recommend” signifies a strong recommendation that should be adopted as routine practice, whether in favor of or against the use of a specific intervention.
- “We suggest” represents a conditional recommendation applicable to most situations; however, due to the lack of robust evidence or expected variation in treatment efficacy, other approaches may be justifiable.

**Table 2:** Implications of the strength of recommendation for clinicians, patients, and policymakers

Intended users	Strong	Conditional
**Policymakers**	The recommendation should be adopted as a health care policy in most situations.	Substantial debate and stakeholder involvement are required.
**Patients**	Most individuals would want the intervention to be indicated and only a small number would not accept this recommendation.	Most individuals would want the intervention to be indicated; however, a considerable number would not accept this recommendation.
**Clinicians**	Most patients should receive the recommended intervention.	The clinician should recognize that different choices will be appropriate for each patient to make a decision consistent with patient values and preferences.

Source: Diretrizes metodológicas: Sistema GRADE – Manual de graduação da qualidade da evidência e força de recomendação para tomada de decisão em saúde/Ministério da Saúde, Secretaria de Ciência, Tecnologia e Insumos Estratégicos, Departamento de Ciência e Tecnologia.. 13

## RESULTS

The presentation of the protocol topics was organized into diagnosis, target population, and therapeutic approach.

### Diagnosis

The clinical protocol advocates that, for the realization and confirmation of the diagnosis, the following should be assessed:

#### Patient history

During the patients' assessment, the precise onset of neurological manifestations and their course (stable versus unstable condition) should be evaluated. A sudden onset of focal neurological deficit indicates the possibility of a stroke. Headache and seizures are more common symptoms in hemorrhagic strokes than in AISs, but confirmation can only be made after imaging. The presence of risk factors for vascular diseases should always be investigated, with systemic arterial hypertension being the most important for both ischemic and hemorrhagic lesions. 14

#### Physical examination

The Prehospital Assessment Scale can be used as a screening method. 15 16

If the patient presents any of the following characteristics, the result is positive. First, facial droop, defined as a noted asymmetry when the patient is asked to smile. Second, arm weakness, observed when the patient is asked to extend the arms forward at a 90° angle to the trunk and hold them in position for 10 seconds, with either one arm not moving or remaining in position compared with the contralateral side. Finally, abnormal speech, noted when the patient is asked to pronounce the phrase “na casa do padeiro nem sempre tem trigo” (“in the baker's house, there isn't always wheat”), the patient utters incomprehensible words, uses incorrect words, or is unable to pronounce the phrase.

Considering that some patients may present focal signs not represented in the Cincinnati scale, the prehospital care team should be trained to recognize other signs of sudden onset, such as vertigo, numbness or tingling in one half of the body, difficulty seeing in one or both eyes, and sudden, intense headaches without an apparent cause. The hospital care team should prioritize the use of the National Institute of Health and Stroke Scale (NIHSS), which is highly valuable for diagnostic, prognostic, and sequential patient assessment.

#### Imaging examination

The most widely used, readily available, and cost-effective imaging method for the initial assessment of AIS is noncontrast computed tomography (NCCT) of the head, which demonstrates early signs of ischemia in up to 67% of cases within the first 3 hours of symptom onset, 17 and in up to 82% of cases within the first 6 hours of ictus (the last known well time). 18 Detection increases to approximately 90% after 1 week. 19 20 Additionally, this method presents good capability to identify intracranial bleeding. Ischemic injury appears as hypodensity (not enhancing with contrast), usually in the territory supplied by the middle cerebral artery.

Magnetic resonance imaging (MRI) is more sensitive and accurate in identifying and locating acute vascular lesions, especially when diffusion/perfusion techniques are used. However, it requires more time for completion, which may be crucial for deciding on thrombolytic treatment. 17 In centers equipped with mechanical thrombectomy capabilities, a CT angiography should be conducted immediately following the NCCT scan to assess for large vessel occlusion. 1 When there is suspicion of pulmonary disease, a chest X-ray is recommended (for patients eligible for thrombolysis or thrombectomy, the X-ray should be conducted after these interventions to save time).

Other complementary tests in the clinical suspicion of stroke, are: resting electrocardiogram (ECG); capillary blood glucose; complete blood count (with platelet count); prothrombin time with international normalized ratio (INR) measurement; activated partial thromboplastin time; and serum levels of potassium, sodium, urea, creatinine, and troponin. These tests should not delay thrombolysis, except for INR in patients using vitamin K antagonists. The ECG aims to identify arrhythmias that increase the risk of stroke, signs of myocardial infarction, or an associated aortic dissection, while blood tests assess the degree of coagulability and situations that may mimic or exacerbate an ongoing stroke (e.g., hypoglycemia, infection, or electrolyte disturbances). 8 Elevated troponin levels can occur in acute strokes, indicating increased severity.

#### Differential diagnosis

The physician's knowledge of the primary forms of onset of cerebral disorders is essential for the clinical diagnosis of hemorrhagic or ischemic stroke. For example, a deficit that develops over weeks is usually due to a mass-effect brain lesion, such as a brain neoplasm or abscess. On the other hand, a subdural hematoma needs to be distinguished from a stroke due to its more prolonged course of focal dysfunctions, typically associated with head traumas. However, the differential diagnosis must be made through neuroimaging (CT or MRI).

Transient ischemic attacks (TIAs) can be confused with aura migraines, characterized by focal signs or symptoms, usually visual, such as scintillating scotomas, hemiparesis, or other focal deficits. 21 Seizures may be confused with TIAs or strokes. Most seizures produce positive motor or sensory activity, while most strokes or TIAs produce negative symptoms.

Sometimes, postictal paresis (Todd's paralysis) or paralysis may occur, lasting from minutes to days. This symptom may have spontaneous recovery and must be differentiated from AIS. The postictal state observed after a seizure can also occur in some ischemic syndromes. A small proportion of strokes (10%), especially embolic ones, are associated with concurrent seizures. Other conditions that may mimic a stroke include hypoglycemia, Ménière's disease, or other peripheral vestibulopathies.

### Treatment

**Figure 1:**
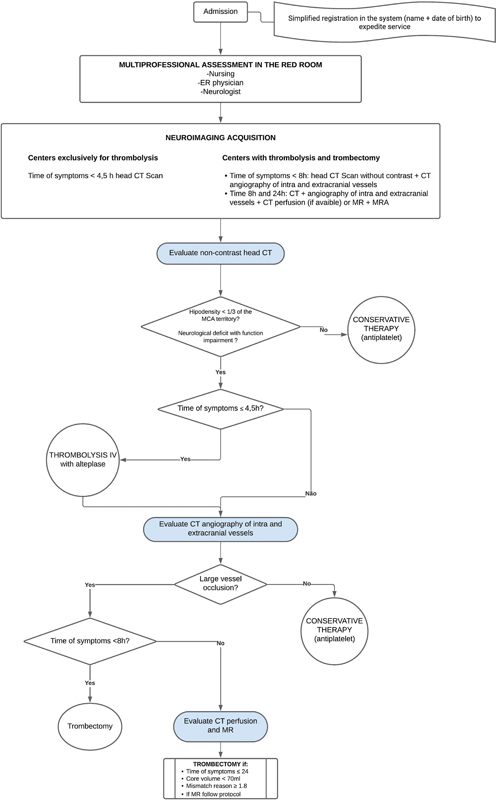
Flowchart for the treatment of patients with suspected acute ischemic stroke (AIS). More details are presented in the clinical protocol official publication. 10

Four recommendations have been incorporated into the SUS, from the MoH's updated AIS protocol, as summarized in Table 3 . Figure 1 provides a detailed overview of the treatment procedures for a patient with suspected AIS within 24 hours of onset, according to the protocol. Here, we present the recommendations, the rationale for decision-making, and, where applicable, the implementation of considerations outlined in the clinical protocol published by the MoH. Detailed information regarding the supporting evidence for each recommendation can be found in the original publication. 10

**Table 3:** Summary of recommendations made by the panel

**Alteplase**	We recommend using alteplase in patients with AIS in any vascular territory with symptom onset up to 4.5 hours (moderate-quality evidence; strong recommendation).
**Mechanical thrombectomy**	We recommend using mechanical thrombectomy in patients with occlusion involving the intracranial internal carotid artery, the first segment of the M1, or both, with symptom onset up to 8 hours (moderate-quality evidence, strong recommendation); provided that the following criteria are met: • NIHSS ≥ 6; • mRS ≤ 2 before the qualifying event.
	We recommend mechanical thrombectomy in patients with a symptom onset window between 8 and 24 hours (moderate-quality evidence, strong recommendation); provided that the following criteria are met: • NIHSS ≥ 6; • mRS ≤ 2 before the qualifying event; • Infarct volume (assessed by perfusion or diffusion): - Less than 21 cm ^3^ (≥ 80 years; NIHSS > 10); - Less than 31 cm ^3^ (< 80 years; NIHSS: 10–19); - Less than 51 cm ^3^ (< 80 years; NIHSS > 20); - Less than 70 cm ^3^ in patients with mismatch > 1.8, penumbra greater than 15 mL, and who present a symptom onset window between 8 and 16 hours.
**General anesthesia or conscious sedation**	We suggest using general anesthesia or conscious sedation in patients eligible for mechanical thrombectomy at the discretion of the responsible team (low certainty of evidence, conditional recommendation).

Abbreviations: AIS, acute ischemic stroke; M1, middle cerebral artery; mRS, modified Rankin scale; NIHSS, National Institutes of Health Stroke Scale. Note: The detailed eligibility criteria for the recommendations are described in the Clinical Practice Guideline (PCDT) and should be followed for implementation. More details are presented in the clinical protocol's official publication. 10

### Alteplase

Thrombolysis is a recommended treatment for AIS, involving the use of thrombolytic medications for clot dissolution, such as alteplase, streptokinase, and tenecteplase. Among these medications, alteplase is the most common and the only one recommended in this protocol.

Recent studies indicate a potential beneficial effect of tenecteplase in patients with AIS. However, there is currently no approved indication for AIS treatment in the product labeling, pending more definitive studies. As for the use of streptokinase, studies evaluating its efficacy and safety did not show favorable results and were prematurely discontinued; 22 there is no evidence to support the treatment of ischemic stroke with this medication.

The effectiveness of therapy in patients with AIS depends on the time elapsed between symptom onset and the initiation of treatment. Due to the short therapeutic window, the number of patients receiving treatment is small, and prevention of adverse outcomes, such as disabilities, is only possible in 6 out of 1,000 stroke patients. 23

#### Recommendation

The MoH recommends using alteplase in patients with AIS in any vascular territory, with symptom onset up to 4.5 hours, according to the eligibility criteria described in the clinical protocol (moderate-quality evidence; strong recommendation).

#### Evidence synthesis

Evidence used to support the recommendation in patients with symptoms up to 4.5 hours.

To address the clinical question, two RCTs 24 25 cited in the previous Clinical Practice Protocol for AIS Thrombolysis 26 were considered, and no additional studies were identified in the subsequent searches. More details regarding the outcome of each stage in the study selection process can be viewed in the official publication of AIS clinical protocols. As the studies had different inclusion criteria, specifically regarding the symptom onset window, up to 3 hours for the National Institute of Neurological Disorders and Stroke rt-PA Stroke Study Group (NINDS), 24 and between 3 and 4.5 hours for the ECASS35 study), 25 meta-analyses were not conducted. Therefore, the results were described narratively, and effect measures were calculated individually.

The primary study evaluating the role of thrombolytics in stroke was conducted by the NINDS and published in 1995. 24 A total of 624 patients were randomized to receive alteplase or placebo within 3 hours of stroke symptom onset. A higher proportion of patients achieved functional independence, with a modified Rankin scale (mRS) between 0 and 1 in the alteplase group (27.2%, 85/312, compared with the placebo group (17%, 53/312; RR: 1.60; 95% CI: 1.18–2.18). Additionally, a higher proportion of patients with ischemic stroke symptoms experienced symptomatic intracranial hemorrhage in the alteplase group, 6% (20/312), compared with 1% (2/312) in the placebo group (RR: 10.00; 95% CI: 2.36–42.42). The study has various inclusion and exclusion criteria, limiting the generalizability of the results.

More recently, Hacke et al. 25 assessed the impact of alteplase in AIS patients when administered within a symptom onset window between 3 and 4.5 hours in a double-blind randomized clinical trial. A total of 418 patients were randomized to the alteplase group and 403 to the placebo group. The mean time between symptom onset and treatment initiation was 3 hours and 59 minutes. A higher proportion of patients who achieved functional independence (mRS: 0–1) was observed in the alteplase group (52%; 219/418) compared with the placebo group (45%; 182/403; RR: 1.16; 95% CI 1.01–1.34). Regarding the safety profile of alteplase, a higher proportion of patients experienced symptomatic intracranial hemorrhage in the alteplase group (8%; 33/418) compared with the placebo group (3%; 14/403; RR: 2.27; 95% CI: 1.23–4.18). An increased risk of any bleeding during the 90-day follow-up was also observed (RR: 1.53; 95% CI: 1.18–2.0). However, there was no difference in mortality outcomes (RR: 0.91; 95% CI: 0.66–1.44) or other adverse events (RR: 1.02; 95% CI: 0.81–1.30).

For the outcomes of functional independence, any intracranial hemorrhage, and mortality, it was deemed that there are some concerns regarding the overall risk of bias in Hacke's study 25 due to the randomization process. For the outcomes of functional independence and symptomatic intracranial hemorrhage, there were some concerns about the overall risk of bias in the NINDS study, 24 due to the randomization process and the selection of reported outcomes. Details regarding the complete assessment of bias, results regarding the assessment of the evidence profile according to the GRADE methodology, and decision-making tables can be found in the PCDT appendix, available on the MoH's website. 10

#### Considerations for implementation

Alteplase is already available in the SUS. The panel of experts who developed this clinical protocol considered that there is evidence of its effectiveness and safety when used in patients with a symptom onset window of less than 4.5 hours. The recommendation development group also indicates the use of alteplase in patients diagnosed with AIS in any vascular territory with symptom onset up to 4.5 hours, according to the clinical eligibility criteria outlined in Table 4 .

**Table 4:** Eligibility criteria for the use of alteplase in patients with acute ischemic stroke with onset of less than 4.5 hours from the beginning of symptoms to medication infusion

	Inclusion criteria	Exclusion criteria 1
	• Assessment by a neurologist to confirm AIS; • CT or MRI without signs of intracranial hemorrhage	Patients presenting any of the contraindications to the procedure, which include: • Spontaneous complete immediate resolution of symptoms; • Early hypodensity area on computed tomography affecting more than one-third of the middle cerebral artery territory; • Major surgery within the last 14 days; • History of intracranial hemorrhage; • Arteriovenous malformations or intracranial tumors; • Systolic blood pressure > 185 mmHg after intravenous antihypertensive treatment; • Diastolic blood pressure > 110 mmHg after intravenous antihypertensive treatment; • Gastrointestinal or genitourinary bleeding in the last 21 days; • Platelet count < 100,000/mm ^3^ ; INR > 1.7; APTT > 40s; • Coagulation defect (INR > 1.7); • Use of direct oral anticoagulants in the last 48 hours, if renal function is normal. If the patient has normal coagulation tests, has stopped the direct anticoagulant for more than 48 hours, and has no other contraindications, thrombolysis can be performed. In patients using dabigatran, if the reversal agent is available (idarucizumab), it can be used, and thrombolysis can be performed shortly after; • Suspicion of subarachnoid hemorrhage, even with a normal tomography; • Therapeutic dose of low molecular weight heparin in the last 24 hours; • Bacterial endocarditis; • Aortic arch dissection; • Active internal bleeding, evidence of active bleeding at a site not amenable to mechanical compression. NOTE 1: The use of antiplatelet therapy and a seizure episode are not contraindications for thrombolysis. Some specific factors may influence the risk-benefit profile of thrombolytic therapy, but they do not constitute absolute contraindications to its use, including NIHSS > 22, age > 80 years, and the combination of prior stroke and diabetes mellitus. The presence of a known unruptured aneurysm is not an exclusion factor for AIS treatment; however, individualization of treatment is recommended, especially for larger aneurysms. NOTE 2: The patient or legal guardian must be informed about the risks and benefits of thrombolytic treatment, and this clarification, as well as agreement to undergo the treatment, should be documented in the medical record. Furthermore, the risk-benefit ratio of thrombolytic treatment should be individually assessed for patients with any of the following relative contraindications: • Mild signs and symptoms; • Previous intracranial surgery, head trauma, or history of severe stroke in the 3 months preceding thrombolytic treatment; • Lumbar puncture within the last 7 days; • History of transmural AMI (with ST-segment elevation) in the last 3 months; • Arterial puncture in a noncompressible site within the last 7 days; • Blood glucose level < 50 mg/dL (contraindication only if the patient completely recovers from symptoms with hypertonic glucose).

Abbreviations: AIS, acute ischemic stroke; AMI, acute myocardial infarction; CT, computed tomography; INR, international normalized ratio; MRI, magnetic resonance imaging; NIHSS, National Institutes of Health Stroke Scale.

Note: More details are presented in the clinical protocol's official publication. 10

Despite the available evidence and the public policies in Brazil, several barriers remain to their full implementation. Currently, 119 hospitals within the SUS are authorized to provide thrombolysis treatment, with 77% of these facilities being located in the South and Southeast regions. The limited organization and shortage of specialists are challenges to expanding access to other regions. Efforts are being made to address these issues, through the implementation of mobile telemedicine, which extends access to underserved areas, including rural regions. Discussion workshops between healthcare managers and specialists are also being used to support the development of new stroke centers. National campaigns to raise awareness about strokes and the urgency of seeking hospital care are essential to increasing the likelihood of patients arriving in time for treatment.

### Thrombectomy

Mechanical thrombectomy is the endovascular procedure for the removal of an obstructive blood clot from a blood vessel, performed during angiography using catheters to guide a device to the vessel that is occluding a cerebral artery. There are two types of devices: a removable self-expanding stent (stent-retriever), which integrates with the clot and is then withdrawn, extracting the clot from circulation; and a suction system that aspirates the clot, clearing the artery.

#### Recommendations

The Brazilian MoH recommends the use of mechanical thrombectomy in patients with occlusion involving the intracranial internal carotid artery, the first segment of the middle cerebral artery (M1), or both, with symptom onset within 8 hours (moderate-quality evidence, strong recommendation). This recommendation is subject to the following criteria being met: NIHSS ≥ 6 and an mRS ≤ 2 before the qualifying stroke.

Similarly, this procedure is also recommended for patients with a symptom onset between 8 and 24 hours (moderate-quality evidence, strong recommendation), who also present with mismatch (presuming the presence of potentially salvageable viable brain tissue).

There are two ways to assess mismatch: first, symptom duration between 8 and 24 hours and clinicoradiological mismatch (a small lesion on imaging with high NIHSS), with stroke volume measured through the core on perfusion CT or diffusion on MRI; eligible patients have a volume of either less than 21 cm ^3^ (≥ 80 years and NIHSS > 10), less than 31 cm ^3^ (< 80 years; NIHSS: 10–19), or less than 51 cm ^3^ (< 80 years; and NIHSS > 20). Secondly, a window of symptoms between 8 and 16 hours and core-perfusion mismatch on perfusion CT or diffusion-perfusion on MRI; eligible patients with a stroke volume (core) less than 70 cm ^3^ , with a mismatch ratio > 1.8, and mismatch volume greater than 15 mL. In both recommendations, the specific eligibility criteria defined in the Clinical Practice Protocol and outlined in Table 2 should be followed.

#### Evidence synthesis

For evidence used to support the recommendation of mechanical thrombectomy in patients with symptom onset up to 8 hours, the expert panel relied on the references and evidence synthesis presented in the CONITEC recommendation report number 58917. 27 Additionally, three systematic reviews with meta-analyses and the Brazilian study RESILIENT 27 were considered in the updated publication. In the synthesis presented, evidence was found showing a significant difference in functional independence at 90 days (mRS: 0–2) in ischemic stroke patients who underwent mechanical thrombectomy (odds ratio [OR]: 2.06; 95% CI: 1.71–2.49; 9 studies; 2,072 participants), compared with those who received the best medical treatment. No difference was detected between the groups regarding the risk of death at 90 days (OR: 0.85; 95% CI: 0.69–1.05). Additionally, no differences were found between the groups regarding the risk of symptomatic intracranial hemorrhage (OR: 1.20; 95% CI: 0.78–1.84). Details regarding the comprehensive assessment of the bias risk of the included studies for this synthesis, results regarding the assessment of the evidence profile according to the GRADE methodology, and decision-making tables can be found in the Clinical Protocol appendix. 10

As for evidence used to support the recommendation of mechanical thrombectomy in patients with symptom onset greater than 8 hours, this clinical protocol presented the references of the two randomized clinical trials and evidence synthesis presented in the recommendation report number 677/2021, 29 related to the proposal for incorporation of mechanical thrombectomy for ischemic stroke with a symptom onset window greater than 8 hours and less than 24 hours.

Additionally, the results of two studies 30 31 evaluating the use of mechanical thrombectomy in ischemic stroke patients with symptom onset times of between 6 and 16 hours and between 6 and 24 hours were considered during the systematic review process used for the AIS clinical protocol update. There was a higher incidence of patients with mRS 0 to 2 when compared with the best clinical management (RR: 3.00; 95% CI: 2.06–4.37), as well as a higher recanalization success rate (RR: 1.95; 95% CI: 1.49–2.54). A lower incidence of patients with neurological deterioration was also observed in the group that underwent mechanical thrombectomy versus placebo (RR: 0.58; 95% CI: 0.36–0.94). However, no difference between the groups was detected for the outcomes: mortality (RR: 0.76; 95% CI: 0.41–1.4); symptomatic intracranial hemorrhage (RR 1.63; 95% CI 0.65–4.06); grouped adverse events (RR: 0.89; 95% CI: 0.74–1.07); and parenchymal hematoma type 2 (RR: 2.61; 95% CI: 0.71–9.52).

More details on the results of the meta-analyses, the complete assessment of the risk of bias of the included studies, the assessment of the evidence profile according to the GRADE methodology, and decision-making tables can be found in the clinical protocol appendix available on the MoH's website. 10

#### Considerations for implementation

Mechanical thrombectomy for a symptom onset window of up to 8 hours has recently been incorporated into SUS, with important scientific evidence of its effectiveness and safety. 28 Its indication was recommended according to the eligibility criteria presented in Table 5 .

**Table 5:** Eligibility criteria for the use of mechanical thrombectomy in patients with acute ischemic stroke

Symptom onset	Inclusion criteria	Exclusion criteria
Within 8 hours or the patient was last seen well (without neurological deficits) within the last 8 hours.	Occlusion involving the intracranial internal carotid artery, the first segment of the M1, or both, that can be treated within 8 hours of symptom onset (defined as the time the patient was last seen in a normal state of health); Prestroke score of 0 or 2 on the mRS; NIHSS score ≥ 6 at presentation.	Patients whose imaging studies show evidence of recent intracranial hemorrhage or large infarction, defined by an ASPECTS of less than 6 (0–10, with higher values indicating a lower infarct burden) or < 5 on diffusion-weighted MRI.
Between 8 and 24 hours or the patient was last seen well (without neurological deficits) between 8 and 24 hours.	Signs and symptoms consistent with the diagnosis of an AVCi; NIHSS ≥ 6; mRS ≤ 2 before the qualifying stroke; Clinical-radiological mismatch with infarct volume (assessed by core on perfusion CT or diffusion on MR): • < 21 cm ^3^ (≥ 80 years; NIHSS > 10); • < 31 cm ^3^ (< 80 years; NIHSS 10–19); • < 51 cm ^3^ (< 80 years; NIHSS > 20); • Mismatch assessed by core-perfusion on perfusion CT or by diffusion and perfusion on MR. Volume < 70 cm ^3^ , mismatch ratio > 1.8, mismatch volume > 15 mL, and symptom onset window 8–16 hours.	Patients meeting any of the following criteria: • > 90 years old; • Allergy to iodine; • Known hereditary or acquired bleeding diathesis, deficiency of coagulation factor; recent oral anticoagulant therapy with INR > 3; • Seizures at stroke onset; • Baseline platelet count < 50,000/uL; • Severe hypertension; • Suspected presumptive septic embolism; • Suspected bacterial endocarditis; • History of bleeding in the last 30 days.

Abbreviations: AIS, acute ischemic stroke; AMI, acute myocardial infarction; ASPECTS, Alberta stroke program early CT score; AVCi, acute anterior circulation ischemic stroke; CT, computed tomography; INR, international normalized ratio; M1, middle cerebral artery; MRI, magnetic resonance imaging; mRS, modified Rankin scale; NIHSS, National Institutes of Health Stroke Scale.

Note: More details are presented in the clinical protocol official publication. 10

Finally, the panel of experts emphasizes the following aspects for the implementation of mechanical thrombectomy: the establishment of a type IV stroke emergency care center, which would include centers qualified to perform thrombectomy, having specific physical, human, and material resources for the procedure. Update of the MoH's stroke document describing the care pathway within the emergency and urgent care network. Discussions on barriers to implementation were mainly discussed by the panel. Challenges in implementation are linked to the limited number of trained professionals for specialized treatment (such as interventional neuroradiologists), insufficient availability of anesthesiologists, inadequate hospital infrastructure, and the absence of well-organized networks to ensure that patients are directed to the appropriate facilities.

Nonetheless, progress has been made in Brazil through policies implemented by the MoH and the support of specialists in training and organizing services. Expanding formal training programs for additional specialists can further enhance access to care.

### General anesthesia or conscious sedation for mechanical thrombectomy

This clinical g protocol recommends either general anesthesia or conscious sedation before mechanical thrombectomy. The choice of anesthetic modality should consider individual aspects (fasting, agitation, and level of consciousness), vomiting, procedure complexity, and duration, among other factors. Strict monitoring of systemic blood pressure is suggested, with narrow pressure intervals, especially during induction in the case of general anesthesia and at the time of vessel opening. Episodes of hypotension and hypertension should be avoided, especially after achieving effective recanalization (modified Thrombolysis in Cerebral Infarction, mTICI), when the target blood pressure should be adjusted to levels below 160/100 mmHg, with experts' opinions suggesting that systolic pressure be reduced to < 140 mmHg to prevent reperfusion bleeding. The team should collectively decide on the target or adjust pressure levels during the procedure.

#### Recommendation

We suggest using general anesthesia or conscious sedation in patients eligible for mechanical thrombectomy at the discretion of the responsible team (low certainty of evidence, conditional recommendation).

#### Evidence used to support the recommendation in patients eligible for mechanical thrombectomy

This clinical protocol 10 included a systematic review comparing the effects of general anesthesia versus conscious sedation in patients eligible for mechanical thrombectomy. The evidence suggests that there is no difference between using general anesthesia versus conscious sedation for outcomes such as any bleeding (RR: 0.89; 95% CI: 0.45–1.75), mortality (RR: 0.77; 95% CI: 0.53–1.13), and intervention-related vascular complications, such as artery dissection or perforation and groin hematoma (RR: 1.18; 95% CI: 0.62–2.26).

Although we were unable to detect statistical differences between general anesthesia and conscious sedation, it seems that patients undergoing general anesthesia have a higher rate of successful recanalization (RR: 1.11; 95% CI: 1.00–1.23) and functional independence (mRS: 0–2) at 3 months (RR: 1.24; 95% CI: 1.00–1.55).

The risk of bias in the included systematic review indicated that it has a low risk of bias. However, all five included studies had a high risk of bias in the domains of allocation concealment and participant blinding, and one study was judged as having a high risk of bias for selective outcome reporting. Additionally, three studies had an unclear risk of bias in the other domains. All details regarding the risk of bias assessment and results regarding the evaluation of the evidence profile according to the GRADE methodology, as well as decision-making tables, can be found in the clinical protocol appendix, available on the MoH website. 10

#### Considerations for implementation

The panel of experts recommends closely monitoring blood pressure values to prevent hypotension during sedation and especially during anesthesia, as well as reducing pressure at the time of vessel opening to avoid reperfusion injury. Additionally, it is advised that the procedure be initiated with conscious sedation and, if necessary, transition to general anesthesia.

### Monitoring

According to the current national protocol, patients who have undergone thrombolytic therapy should observe dietary restrictions (no food or liquids, including orally administered medications) for 24 hours. Before the first meal, all stroke patients—whether they are undergoing conservative treatment, thrombolysis, or thrombectomy—should be screened for dysphagia to prevent poststroke pneumonia and reduce the risk of early mortality. Swallowing tests with water or multiple consistency tests are recommended during dysphagia assessment.

## DISCUSSION

The aim of the publication of the new MoH protocol was to provide a comprehensive and updated set of recommendations for adult patients with AIS treated within the Brazilian SUS. In addition to the pre-existing use of alteplase, new recommendations for thrombectomy and anesthesia/sedation were incorporated. It is noteworthy that the technologies outlined in the current protocol align with major international guidelines. 1 22 Four recommendations were formulated, with the majority supporting the use of the evaluated technologies.

In the current clinical protocol, the use of mechanical thrombectomy is recommended for patients with large vessel lesions and symptom onset within 8 hours or between 8 and 24 hours. We did not cover the recent evidence on thrombectomy in cases of basilar artery occlusion, as the relevant studies were published after the completion of the panel's work, and therefore this issue was not evaluated. Alteplase is recommended for eligible patients with symptoms within 4.5 hours, including those indicated for thrombectomy.

The international guideline from the American heart association (AHA) also recommends the same technologies indicated in this Brazilian clinical protocol for patients with AIS. 1 According to this AHA guideline, mechanical thrombectomy is recommended within 6 hours of symptom onset for selected patients based on CT and angiography, and for patients with symptom onset within 6 to 24 hours who meet specific criteria from the DAWN and DEFUSE 3 studies, 29 30 although the recommendation for them is considered reasonable. In this Brazilian protocol, 10 we recommend thrombectomy up to 8 hours after symptom onset for selected patients based only on CT and CT, as the national RESILIENT study 27 demonstrated the benefit of the procedure in this timeframe and was published after the American guidelines. Regarding alteplase use, the AHA guideline recommends alteplase within a 3-hour window and from 3 to 4.5 hours. These findings demonstrate that this protocol is in line with international recommendations.

The use of tenecteplase as an alternative for thrombolysis was also a clinical question addressed by this protocol. However, because it does not have an approved indication by the Brazilian regulatory agency, ANVISA, as of the time of this update, the current Brazilian protocol does not recommend its use as an alternative to thrombolysis in patients with AIS. This was the only recommendation not in line with international guidelines, which recommend the use of tenecteplase as an option for thrombolysis in patients eligible for mechanical thrombectomy. Additionally, it can be considered as an alternative to alteplase in patients with neurological impairment and without significant intracranial occlusion.

## CONCLUSION

With the publication of the protocol, the Brazilian MoH aims to guide the management of patients with AIS. 10 In addition to the evidence available in the scientific literature, the recommendations considered relevant aspects for the Brazilian context, such as the availability of medications, the acceptability of interventions by the population and healthcare professionals, and associated costs. This documentation was developed considering the need for comprehensive recommendations and the perspective of different medical specialties. It is important to emphasize the need for future updates as new evidence emerges.
