# Erratum

**DOI:** 10.1055/s-0045-1810451

**Published:** 2025-07-28

In the article “ Brazilian Public Health System protocol for the diagnosis, treatment, and clinical monitoring of acute ischemic stroke ”, published in the Arq. Neuro-Psiquiatr. 2025;83(5):s00451806923,

Where it reads:

Ana Carolina Pereira Numes Pinto

It should be:

Ana Carolina Pereira Nunes Pinto

